# Analysis of morphological variables and arterialization in the differential diagnosis of hepatic nodules in explanted cirrhotic livers

**DOI:** 10.1186/1746-1596-2-51

**Published:** 2007-12-21

**Authors:** Cristina Nascimento, Adriana Bottino, Cristiane Nogueira, Vera Pannain

**Affiliations:** 1 Department of Pathology, HUCFF, Federal University of Rio de Janeiro, Rodolpho Paulo Rocco av, Rio de Janeiro, Brazil; 2 Department of Internal Medicine, HUCFF, Federal University of Rio de Janeiro, Rodolpho Paulo Rocco av, Rio de Janeiro, Brazil

## Abstract

**Background:**

Many terminologies have been given to dysplastic hepatocellular nodules, which are preneoplastic lesions. In 1995, the International Working Party meeting established the nomenclature and morphological criteria for hepatocellular nodular lesions. Nevertheless, an unequivocal differential diagnosis is sometimes difficult, particularly among large regenerative nodules, dysplastic nodules and hepatocellular carcinoma. Angiogenesis is observed during hepatocarcinogenesis and the presence of the isolated arteries may help to discriminate these nodules. The relevance of the International Working Party histological variables and presence of the isolated arteries were analyzed with regard to the diagnosis of large regenerative nodules, low and high grade dysplastic nodules and hepatocellular carcinoma, in order to evaluate which have a real contribution in such diagnoses.

**Methods:**

One hundred and seven nodular hepatocellular lesions over 5 mm (or smaller nodules with a different color) from explanted cirrhotic livers were analyzed and classified following the criteria of the International Working Party. Classifications were as follows: large regenerative nodules, low grade dysplastic nodules, high grade dysplastic nodules and hepatocellular carcinoma. The presence of isolated arteries (not related to the portal tracts or fibrosis) was verified for the nodules.

**Results:**

Among the 107 nodular lesions studied, 17 were classified as large regenerative nodules, 38 as low grade dysplastic nodules, 28 as high grade dysplastic nodules and 24 as hepatocellular carcinoma. The most relevant International Working Party variables in the differential diagnosis of the nodules were cellularity, trabeculae thickness, cytoplasmic staining, nuclear atypia, pseudoacinar pattern, portal tracts, nucleocytoplasmic ratio and mitosis. The isolated arteries, identified by hematoxylin and eosin staining, were important discriminating between two groups: low grade lesions (large regenerative nodules/low grade dysplastic nodules) and high grade lesions (high grade dysplastic nodules/hepatocellular carcinoma) (P < 0.001).

**Conclusion:**

The International Working Party criteria allow for the classification of the majority of hepatocellular nodules. However, other features such as cytoplasmatic tintorial affinity and pseudoacinar pattern may contribute to these diagnoses. The finding of isolated arteries in a nodular lesion should be investigated carefully, since the nodule could be a dysplastic lesion or hepatocellular carcinoma.

## Background

Hepatic carcinogenesis may occur in a multistep process characterized by genetic and epigenetic alterations as well as in clonal expansion of liver cells over years of time [1]. Preneoplastic lesions are considered to result from a continuous process in which the hepatocyte clones form specific foci and dysplastic nodules that can progress to early and advanced hepatocellular carcinoma (HCC) [2,3]. This neoplasia occurs in chronic liver disease, mainly in cirrhotic livers, and it has been noted that 20% of cirrhotic patients who die as a result of liver disease show HCC in autopsy [4]. It was also observed that patients who underwent a liver transplantation showed incidental HCC in the explanted liver [5].

Preneoplastic nodules are represented by a wide variety of terminologies in the literature [6-11]. The fact that the different criteria used for the diagnoses are not always equivalent or easily superimposed makes the comparison between studies difficult. In 1995, a consensus regarding the characterization and nomenclature of hepatocellular nodular lesions took place at the International Working Party (IWP) [6]. At that time, pathologists and hepatologists established morphological criteria to classify these lesions. In that meeting, the preneoplastic nodules were defined as low grade dysplastic nodules (LGDN) and high grade dysplastic nodules (HGDN), and regenerative nodules that measured over 5 mm diameter were named large regenerative nodules (LRN).

Improvements in imaging techniques have increased the frequency of detection of nodular lesions in cirrhotic livers, but frequently the findings are nonspecific and it is necessary to perform pathological examination to obtain the correct diagnosis. Nevertheless, reaching an unequivocal diagnosis is sometimes difficult, even for expert liver pathologists.

Structural and functional changes in the vascular architecture occur early during hepatic carcinogenesis [12]. Isolated arteries in dysplastic nodules are considered a factor indicative of a neoplastic process, and they may help to discriminate between large regenerative nodules (LRN) and dysplastic nodules [12].

In an attempt to establish parameters that could help the general pathologist in their daily practice, we analyzed the relevance of both the histologic variables proposed by IWP and isolated arteries for the differential diagnosis among LRN, dysplastic nodules (LGDN, HGDN) and HCC.

## Methods

A cross-sectional study was performed in explanted livers from cirrhotic patients at the University Hospital of the Federal University of Rio de Janeiro, Brazil. The study was approved by the local Ethic's Committee (HUCFF, CEP: 009/005) and the collected data were kept in a database. Nodules that measured over 5 mm in diameter or those which had a different color compared to the adjacent liver parenchyma were studied and classified as LRN, LGDN, HGDN and HCC according to the IWP consensus [6]. The smaller nodules were also included since they presented microscopic characteristics that allowed a diagnosis of dysplastic nodule or HCC.

For the classification of the different nodules, the following morphological IWP variables were studied: cellularity, thickness of hepatocellular plates, nuclear atypia (pleomorphism and irregular contour), nucleocytoplasmatic ratio, cytoplasmic staining (eosinophilic, basophilic, amphophilic and clear), pseudoacinar pattern, stromal invasion, portal tracts, biliary pigment, lipid vacuoles, Mallory hyaline and nuclear glycogen. The number of mitoses was counted using a 10 high-field power (HPF) microscope. Cellularity was considered low when it was possible to add a hepatocyte nucleus between two hepatocytes, and high when it was not possible to accomplish this procedure. When an intermediate pattern between low and high cellularity was observed, the cellularity was defined as moderate.

Briefly, according to the IWP guidelines, LRN are composed of hepatocytes similar to those in adjacent parenchyma, trabeculae are one or two hepatocytes wide, portal tracts are normal or scarred and mitotic activity is absent. In LGDN, hepatocytes are minimally abnormal, the nuclear-cytoplasmic ratio is normal or slightly increased, nuclear atypia is minimal, mitotic activity is absent and the portal tract is present. HGDN have any of the LGDN features, the nuclear-cytoplasmatic ratio is high, nuclear atypia is more pronounced, cytoplasm basophilia, liver plates are more than two cells thick and mitosis may be present. In HCC, cell size is usually decreased, nuclear density is at least twice that of normal, nuclear atypia is more definite, mitotic figures are present and there is pseudoacinar formation. Stromal and portal tract invasion may also be found.

We also studied the presence or absence of the isolated arteries anywhere in the nodules that was not related to the portal tracts or fibrosis area, as identified by hematoxylin and eosin staining (HE). The isolated arteries were defined at a minimum diameter ratio of more than 1/10 of the thickness of the medial layer [12].

The statistical analysis was performed using the SPSS v.11 Program for Windows. The Chi-square test was applied to assess the significance of the association between the nodules and its categorical variables. A significance level of 0.05 was adopted.

## Results

**Figure 1:**
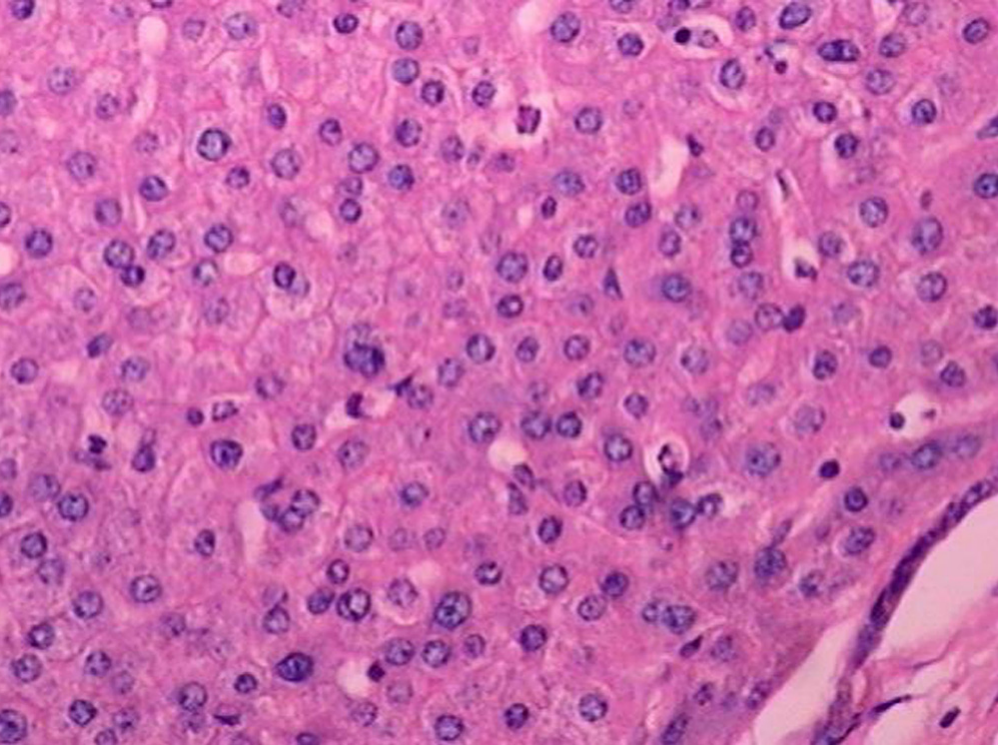
HGDN with high cellularity, pseudoacinar pattern. H&E. Original magnification ×100.

From 2000 to 2004, 107 nodules were studied in 36 explanted livers. Hepatitis C infection was the most prevalent etiology (78%). Nodules size had mean size of 0.8 ± 0.4 cm with a median of 0.7 cm. Seventeen nodules were classified as LRN, 38 as LGDN, 28 as HGDN and 24 as HCC. The medium size of LRN was 0.8 cm (SD = 0.10 cm), the LGDN was 0.7 cm (SD = 0.59 cm), the HGDN and the HCC were 0.9 cm (SD = 0.49 cm) and 0.8 cm (SD = 0.47 cm), respectively. We did not observe a statistically significant association among the size of the different nodules (p = 0.538). A low cellularity was observed in 100% of LRN and the cellularity was similar to the adjacent liver parenchyma. The LGDN showed low or moderate cellularity in the majority of cases, whereas a high cellularity was predominant in HGDN (Figure 1). In the HCC nodules, a high cellularity was observed in 100% of cases (Table 1). Most of the LRN and LGDN showed a thickness of one or two cells in the trabeculae. The HGDN showed two or more cell thickness in all cases (Figure 1), while HCC showed a thickness of three or more cells in most of cases (Table 2). With respect to cytoplasmic staining, LRN displayed a preponderance of eosinophilic cytoplasm (59%) with a tendency to lose this eosinophilic pattern, thus acquiring a similar cytoplasmic staining signature as LGDN; basophilia was prevalent in 46% of HGDN and in 92% of HCC. Hypercromasia and nuclear atypia were also observed, mainly in HGDN and in HCC (Table 3), as well a pseudoacinar pattern (Figure 1, Table 4). The nucleocytoplasmatic ratio was increased specially in HCC (Table 3). Portal tracts were observed in different proportions in LRN, LGDN and HGDN, but they were absent in HCC (Table 4). Mitosis was observed mainly in HCC and stromal invasion was exclusive of this neoplasia. In this study, the most relevant histological criteria for the classification of the nodules were cellularity, thickness of liver plates, cytoplasm staining, nuclear atypia, nuclear hypercromasia, pseudoacinar pattern, portal tracts, nucleocytoplasmatic ratio and mitosis/HPF, while other criteria such as biliary pigment, lipid vacuoles, Mallory's hyaline and nuclear glycogen were not relevant in the differential diagnosis of hepatic nodules, because they were uniformly observed in all nodules.

**Table 1:** Celullarity

Nodules	Low	Moderate	High
LRN	17/17 (100%)	0	0
LGDN	30/38(78.9%)	8/38 (21.1%)	0
HGDN	1/28 (3.6%)	10/28 (35.7%)	17/28 (60.7%)
HCC	0	0	24/24 (100%)

LRN – large regenerative nodules; LGDN – low grade dysplastic nodules; HGDN – high grade dysplastic nodules; HCC – hepatocellular carcinoma.

**Table 2:** Plates cells thickness

Nodules	One	Two	Three or more
LRN	8/17 (47.1%)	9/17 (52.9%)	0
LGDN	6/38 (15.8%)	31/38 (81.6%)	1/38 (2.6%)
HGDN	0	14/28 (50%)	14/28 (50%)
HCC	0	1/24 (4.2%)	23/24 (95.8%)

LRN – large regenerative nodules; LGDN – low grade dysplastic nodules; HGDN – high grade dysplastic nodules; HCC – hepatocellular carcinoma.

**Table 3:** Nuclear hyperchromasia, Nuclear atypia and Nucleocytoplasmic ratio

	Nuclear hyperchromasia	Nuclear atypia	Nucleocytoplasmic ratio
Nodules	Present	Present	High

LRN	2/17 (11.8%)	1/17 (5.9%)	3/17(17.6%)
LGDN	4/38 (10.5%)	13/38 (34.2%)	9/38 (23.7%)
HGDN	9/28 (32.1%)	11/28 (39.3%)	11/28 (39.3%)
HCC	18/24 (75%)	22/24 (91.7%)	21/24(87.5%)

LRN – large regenerative nodules; LGDN – low grade dysplastic nodules; HGDN – high grade dysplastic nodules; HCC – hepatocellular carcinoma.

**Table 4:** Portal tracts and Pseudoacinar pattern

	Portal tracts	Pseudoacinar pattern
Nodules	Present	Present

LRN	17/17(100%)	1/17 (5.9%)
LGDN	25/38 (65.8%)	4/38 (10.5%)
HGDN	14/28 (50%)	11/28 (39.3%)
HCC	0/24	20/24 (83.3%)

LRN – large regenerative nodules; LGDN – low grade dysplastic nodules; HGDN – high grade dysplastic nodules; HCC – hepatocellular carcinoma.

**Figure 2:**
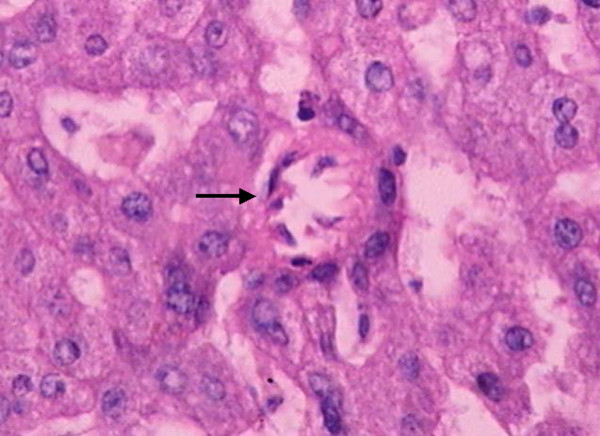
HGDN with isolated artery. H&E Original magnification ×200.

Isolated arteries (Figure 2) were identified with a greater prevalence in HGDN and in HCC (Table 5), although statistical significance was not obtained. Yet, when LRN and LGDN are combined and analyzed together with HGDN and HCC, the presence of an isolated artery was an important characteristic for the differential diagnosis. The frequency of the association was 21.7% in the first group (LRN/LGDN) and 78.2% in the second group (HGDN/HCC) (p < 0.001).

**Table 5:** Isolated artery

Nodules	Present	Absent	Total
LRN	3 (6,5%)	14 (23%)	17
LGDN	7 (15,2%)	31 (50,8%)	38
HGDN	18 (39,1%)	10 (16,4%)	28
HCC	18 (39,1%)	6 (9,8%)	24
Total	46 (100%)	61 (100%)	107

LRN – large regenerative nodules; LGDN – low grade dysplastic nodules; HGDN – high grade dysplastic nodules; HCC – hepatocellular carcinoma.

## Discussion

Until 1995, a number of redundant nomenclatures and conflicting descriptions of the morphological characteristics for dysplastic lesions were responsible for frequent difficulties in diagnoses of these lesions. A correct diagnosis is crucial because each lesion requires a different clinical approach. It has already been suggested that in LGDN the best approach is to perform a biopsy when the lesion's appearance changes with imaging methods. However, in HGDN the management varies in different centers. Some perform a surgical resection when the diagnosis is associated with HCC. Ethanol injection or thermoablation can also be a therapeutic option.

Based on IWP classification, we analyzed 107 nodular lesions from explanted cirrhotic livers. This has been done before by other authors [10,12-14], but not in our Latin American setting. In our study, 17 nodular lesions were classified as LRN, 38 LGDN, 28 HGDN and 24 HCC. With regard to the IWP criteria that consider dysplastic nodules larger than 0.1 cm, the smallest dysplastic nodule identified in our study measured 0.18 cm. When smaller than 0.1 cm, the nodules are called dysplastic foci. Maybe such small lesions are not capable of compromise in patient prognosis, but we should evaluate if these patients should have a closer follow-up. Theise *et al.*[11] also considered that these lesions should be described as dysplastic since nodule size is merely reflection of how clonal expansion has been occurring.

The IWP [6] considers morphological variables in order to achieve the microscopic characterization of these nodular lesions. In our study, the most relevant variable was cellularity, which was smaller in LRN and LGDN and larger in those of HGDN and HCC. Trabeculae thickness also increased as the lesions advanced through the different steps of hepatocarcinogenesis. Regarding cytoplasm tintorial affinity, those defined as HGDN and HCC showed a greater basophilic aspect when compared to LRN and LGDN. Hypercromasia and nuclear atypia were also observed in HGDN and HCC.

All variables described above with the exception of cytoplasmic basophilia were considered as major criteria in the IWP diagnosis of nodular liver lesions, as observed in this study. Some other characteristics also important in their differential diagnosis were pseudoacinar pattern, which was more frequent in HGDN and HCC, and mitosis, observed almost exclusively in HCC lesions. Although these findings were considered as secondary in the IWP, they were helpful in the diagnosis of the different nodules when analyzed with the other characteristics.

Amongst all the morphological characteristics observed for the diagnostic definition, the clear-cut increase in cellularity helped in the discrimination of the different lesions, even using a subjective method for definition. In previous studies the counting of hepatocytes/mm^2 ^had been performed and the final results were similar to ours [15,16]. Perhaps, in the near future, studies using image automatization systems will provide better results [17]. However, we believe that this criterion should not be considered apart from the others in the differential diagnosis of these nodules.

Although the IWP consensus is known and largely adopted by many authors [10,12,18], studies that test its reproducibility and its prospective validation are lacking. Some proposed microscopic criteria are very subjective and lead to great difficulty in differential diagnosis between lesions. Le Bail *et al.*[16] also suggest that the differential diagnosis of benign, atypical or malignant nodular hepatic lesions may even be difficult for highly qualified pathologists. Thus, we believe that the time has come for a critical analysis of the IWP proposal, with an accompanying redefinition of some criteria, once international use by expert hepatopathologists over several years bears enough evidence to support these changes. However, we should consider the possibility that some lesions will not have their biological behavior defined on the basis of microscopic criteria alone, similar to the situation observed in HGDN and HCC. Molecular and immunohistochemical methods may offer collaborative evidence and have been shown as an important contribution to diagnosis [19].

Some studies have suggested that cirrhosis progression from dysplastic nodules to HCC is accompanied by an abnormal vascularization process [12-14]. The number of isolated arteries has also disclosed some abnormalities in a semi-quantitative evaluation in preneoplastic lesions and in HCC [20,21]. Terada *et al.*[21] demonstrated that these arteries are lacking in the normal liver parenchyma and are scarce in chronic hepatitis as well as in LRN. This observation led us to suggest that perhaps these lesions are not dysplastic lesions, and are different from the borderline nodules that contained many arterial elements. In our study, we observed isolated arteries with the HE technique in the different types of nodule, but they were more prominent in those classified as HGDN and HCC. The immunohistochemical study with the use of anti-smooth muscle antibodies may facilitate the identification of arteries in these nodules, allowing a semi-quantitative evaluation that could provide a more objective approach in the differential diagnosis of these types of lesions [12]. A more prevalent artery supply also facilities the angiographic diagnosis of HCC and differentiated non-neoplastic and preneoplastic conditions.

## Conclusion

We conclude that the more relevant criteria in IWP for the differential diagnosis of LRN, LGDN, HGDN and HCC are cellularity, trabeculae thickness, nuclear atypia, mitosis, cytoplasmatic tintorial affinity and pseudoacinar pattern. However, we believe that the presence of isolated arteries in a nodular cirrhotic liver lesion should be investigated carefully, because this may indicate the presence of a dysplastic nodule and, most likely, HCC.

## List of abbreviations

International Working Party (IWP); large regenerative nodules (LRN); low grade dysplastic nodules (LGDN); high grade dysplastic nodules (HGDN); hepatocellular carcinoma (HCC).

## Competing interests

The author(s) declare that they have no competing interests.

## Authors' contributions

CMN evaluated the HE staining, performed analyses and interpretation of the data, and drafted the manuscript; CVN revised the manuscript; and ACB and VLP were responsible for the design and development of the project, as well as revision of the manuscript. All authors read and approved the final manuscript.
